# 2-Anilino-3-(2-hy­droxy­phen­yl)quinazolin-4(3*H*)-one methanol monosolvate

**DOI:** 10.1107/S1600536810030631

**Published:** 2010-08-11

**Authors:** Bi Liu, Xiao-Bao Chen, Xu-Hong Yang, Dong-Feng Pan, Jun-Kai Ma

**Affiliations:** a Faculty of Chemistry and Life Science, Xianning University, Xianning 437100, Hubei, People’s Republic of China; b Institute of Medicinal Chemistry, Hubei Medical University, Shiyan 442000, Hubei, People’s Republic of China; c Department of Oncology, Renmin Hospital, Hubei Medical University, Shiyan 442000, Hubei, People’s Republic of China

## Abstract

In the title compound, C_20_H_15_N_3_O_2_·CH_3_OH, the quinazolin­one ring system is approximately planar, the dihedral angle between the pyrimidinone ring and the adjacent benzene ring being 1.73 (6)°. The pyrimidinone ring makes dihedral angles of 77.58 (6) and 29.62 (6)°, respectively, with the hy­droxy­phenyl and phenyl rings. In the crystal, the components are connected by O—H⋯O and C—H⋯O hydrogen bonds, forming a zigzag chain along the *b* axis.

## Related literature

For the biological activity of quinazoline-4(3*H*)-one derivatives, see: Pandeya *et al.* (1999 ▶); Shiba *et al.* (1997 ▶); Malamas & Millen (1991 ▶); Mannschreck *et al.* (1984 ▶); Kung *et al.* (1999 ▶); Bartroli *et al.* (1998 ▶); Palmer *et al.* (1997 ▶); Tsou *et al.* (2001 ▶); Matsuno *et al.* (2002 ▶). For the synthesis of the title compound, see: Yang *et al.* (2008 ▶).
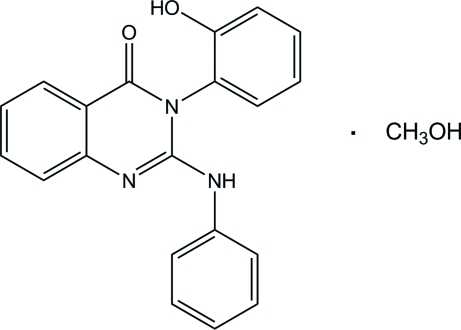


## Experimental

### 

#### Crystal data

C_20_H_15_N_3_O_2_·CH_4_O
*M* *_r_* = 361.39
Monoclinic, 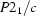

*a* = 11.5575 (18) Å
*b* = 8.7305 (13) Å
*c* = 18.892 (3) Å
β = 106.251 (2)°
*V* = 1830.1 (5) Å^3^
*Z* = 4
Mo *K*α radiation
μ = 0.09 mm^−1^
*T* = 298 K
0.16 × 0.12 × 0.10 mm

#### Data collection

Bruker SMART APEX CCD area-detector diffractometer
Absorption correction: multi-scan (*SADABS*; Sheldrick, 2001 ▶) *T*_min_ = 0.986, *T*_max_ = 0.991
21942 measured reflections
4541 independent reflections
3087 reflections with *I* > 2σ(*I*)
*R*_int_ = 0.074

#### Refinement

*R*[*F* ^2^ > 2σ(*F* ^2^)] = 0.043
*wR*(*F* ^2^) = 0.123
*S* = 1.02
4541 reflections
254 parameters
H atoms treated by a mixture of independent and constrained refinement
Δρ_max_ = 0.15 e Å^−3^
Δρ_min_ = −0.20 e Å^−3^

### 

Data collection: *SMART* (Bruker, 2000 ▶); cell refinement: *SAINT* (Bruker, 2000 ▶); data reduction: *SAINT*; program(s) used to solve structure: *SHELXS97* (Sheldrick, 2008 ▶); program(s) used to refine structure: *SHELXL97* (Sheldrick, 2008 ▶); molecular graphics: *SHELXTL* (Sheldrick, 2008 ▶); software used to prepare material for publication: *SHELXTL*.

## Supplementary Material

Crystal structure: contains datablocks global, I. DOI: 10.1107/S1600536810030631/is2582sup1.cif

Structure factors: contains datablocks I. DOI: 10.1107/S1600536810030631/is2582Isup2.hkl

Additional supplementary materials:  crystallographic information; 3D view; checkCIF report

## Figures and Tables

**Table 1:** Hydrogen-bond geometry (Å, °)

*D*—H⋯*A*	*D*—H	H⋯*A*	*D*⋯*A*	*D*—H⋯*A*
O2—H2*A*⋯O3^i^	0.940 (19)	1.74 (2)	2.6775 (14)	173.8 (17)
C11—H11⋯O1^i^	0.93	2.59	3.3781 (17)	143
O3—H3*B*⋯O1	0.90 (2)	1.85 (2)	2.7237 (14)	164.1 (18)

Symmetry code: (i) 
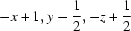
.

**Fig. 1.**
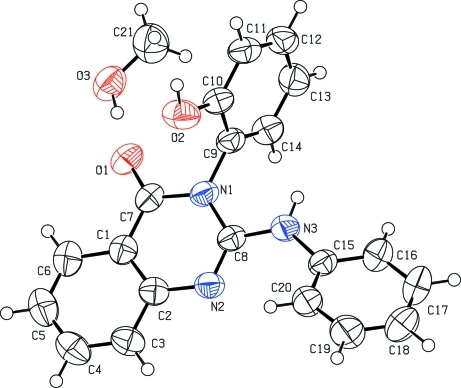
View of the molecular structure of (I), showing the atom labelling scheme and with displacement ellipsoids drawn at the 50% probability level.

**Fig. 2.**
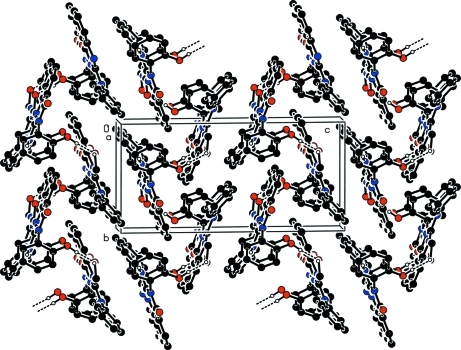
A partial packing view of the crystal packing of (I), showing the formation of O—H···O and C—H···O hydrogen-bonds as dashed lines.

## supplementary crystallographic information

### Comment

Quinazoline-4(3*H*)-one derivatives have numerous biological properties. Some of these activities include antimicrobial (Pandeya *et al.*, 1999; Shiba *et al.*, 1997), antidiabetic (Malamas & Millen, 1991), anticonvulsant (Mannschreck *et al.*, 1984), antibacterial (Kung *et al.*, 1999), antifungal (Bartroli *et al.*, 1998), protein tyrosine kinase inhibitors (Palmer *et al.*, 1997), EGFR inhibitors (Tsou *et al.*, 2001) and PDGFR phosphorylation inhibitors (Matsuno *et al.*, 2002). We have recently focused on the synthesis of heterocyclic compounds using an aza-Wittig reaction. We present here the crystal structure of the title compound, (I) (Fig. 1), which can be used as a precursor for obtaining bioactive molecules.

In the crystal structure, the pyrimidinone heterocycle and the adjacent benzene ring are not planar, but inclined at 1.73 (6)°. Significant intermolecular O—H···O and C—H···O and intramolecular O—H···O contribute strongly to the stability of the molecular configuration (Table 1 and Fig. 2).

### Experimental

The title compound was prepared according to the literature method of Yang *et al.* (2008). To a solution of iminophosphorane (1.40 g, 3.0 mmol) in anhydrous THF (10 ml) was added isocyanatobenzene (3 mmol) under nitrogen at room temperature. After reaction, the mixture was allowed to stand for 10 h at 273–278 K, the solvent was removed under reduced pressure and diethyl ether/petroleum ether (1:2 *v*/*v*, 20 ml) was added to precipitate triphenylphosphine oxide. After filtration, the solvent was removed to give 1-phenyl- 3-(2-ethoxycarbonylphenyl) carbodiimide, which was used directly without further purification. To a solution of 1-phenyl- 3-(2-ethoxycarbonylphenyl) carbodiimide in THF (15 ml) was added 2-aminophenol (3 mmol). After the reaction mixture was allowed to stand for 0.5 h, the solvent was removed and anhydrous ethanol (10 ml) with several drops of EtONa in EtOH was added. The mixture was stirred for 2 h at room temperature. The solution was concentrated under reduced pressure and the residue was recrystallized from ethanol to give the title compound, (I). The product was recrystallized from methanol-dichloromethane (1:1 *v*/*v*, 20 ml) at room temperature to give crystals suitable for X-ray diffraction (yield 85%).

### Refinement

All C-bound H atoms were located at their ideal positions with C—H = 0.93 Å (aromatic) and 0.96 Å (methyl), and refined as riding, with *U*_iso_(H) = 1.2*U*_eq_(C) for aromatic and 1.5*U*_eq_(C) for methyl H atoms. H atoms bonded to N and O atoms were found in a difference map and then refined with distance restraints of N—H = 0.85 (2) Å and O—H = 0.90 (2) Å. The *U*_iso_(H) values were set k times of their carrier atoms (k = 1.2 for N and 1.5 for O atoms).

### Crystal data

**Table:**

C_20_H_15_N_3_O_2_·CH_4_O	*F*(000) = 760
*M**_r_* = 361.39	*D*_x_ = 1.312 Mg m^−^^3^
Monoclinic, *P*2_1_/*c*	Mo *K*α radiation, λ = 0.71073 Å
Hall symbol: -P 2ybc	Cell parameters from 6031 reflections
*a* = 11.5575 (18) Å	θ = 2.5–24.8°
*b* = 8.7305 (13) Å	µ = 0.09 mm^−^^1^
*c* = 18.892 (3) Å	*T* = 298 K
β = 106.251 (2)°	Block, colorless
*V* = 1830.1 (5) Å^3^	0.16 × 0.12 × 0.10 mm
*Z* = 4	

### Data collection

**Table:**

Bruker SMART APEX CCD area-detector diffractometer	4541 independent reflections
Radiation source: fine-focus sealed tube	3087 reflections with *I* > 2σ(*I*)
graphite	*R*_int_ = 0.074
φ and ω scans	θ_max_ = 28.3°, θ_min_ = 1.8°
Absorption correction: multi-scan (*SADABS*; Sheldrick, 2001)	*h* = −15→15
*T*_min_ = 0.986, *T*_max_ = 0.991	*k* = −11→11
21942 measured reflections	*l* = −25→25

### Refinement

**Table:**

Refinement on *F*^2^	Primary atom site location: structure-invariant direct methods
Least-squares matrix: full	Secondary atom site location: difference Fourier map
*R*[*F*^2^ > 2σ(*F*^2^)] = 0.043	Hydrogen site location: inferred from neighbouring sites
*wR*(*F*^2^) = 0.123	H atoms treated by a mixture of independent and constrained refinement
*S* = 1.02	*w* = 1/[σ^2^(*F*_o_^2^) + (0.0641*P*)^2^] where *P* = (*F*_o_^2^ + 2*F*_c_^2^)/3
4541 reflections	(Δ/σ)_max_ = 0.001
254 parameters	Δρ_max_ = 0.15 e Å^−^^3^
0 restraints	Δρ_min_ = −0.20 e Å^−^^3^

### Special details

**Table:**

Geometry. All e.s.d.'s (except the e.s.d. in the dihedral angle between two l.s. planes) are estimated using the full covariance matrix. The cell e.s.d.'s are taken into account individually in the estimation of e.s.d.'s in distances, angles and torsion angles; correlations between e.s.d.'s in cell parameters are only used when they are defined by crystal symmetry. An approximate (isotropic) treatment of cell e.s.d.'s is used for estimating e.s.d.'s involving l.s. planes.
Refinement. Refinement of *F*^2^ against ALL reflections. The weighted *R*-factor *wR* and goodness of fit *S* are based on *F*^2^, conventional *R*-factors *R* are based on *F*, with *F* set to zero for negative *F*^2^. The threshold expression of *F*^2^ > σ(*F*^2^) is used only for calculating *R*-factors(gt) *etc*. and is not relevant to the choice of reflections for refinement. *R*-factors based on *F*^2^ are statistically about twice as large as those based on *F*, and *R*- factors based on ALL data will be even larger.

### Fractional atomic coordinates and isotropic or equivalent isotropic displacement parameters (Å^2^)

**Table:**

	*x*	*y*	*z*	*U*_iso_*/*U*_eq_	
C1	0.14579 (11)	0.79335 (13)	0.16728 (6)	0.0498 (3)	
C2	0.02576 (10)	0.74840 (13)	0.15172 (6)	0.0489 (3)	
C3	−0.05802 (12)	0.85206 (15)	0.16547 (8)	0.0596 (3)	
H3	−0.1387	0.8243	0.1554	0.072*	
C4	−0.02146 (14)	0.99419 (16)	0.19373 (8)	0.0689 (4)	
H4	−0.0774	1.0612	0.2037	0.083*	
C5	0.09758 (14)	1.03937 (16)	0.20762 (8)	0.0704 (4)	
H5	0.1208	1.1371	0.2257	0.085*	
C6	0.18058 (12)	0.94065 (15)	0.19480 (7)	0.0631 (4)	
H6	0.2606	0.9709	0.2043	0.076*	
C7	0.23377 (10)	0.68658 (14)	0.15427 (7)	0.0502 (3)	
C8	0.06580 (9)	0.51041 (13)	0.11378 (6)	0.0463 (3)	
C9	0.27399 (9)	0.42459 (14)	0.12439 (6)	0.0476 (3)	
C10	0.33853 (10)	0.35392 (14)	0.18934 (7)	0.0511 (3)	
C11	0.41817 (10)	0.23803 (15)	0.18518 (8)	0.0579 (3)	
H11	0.4634	0.1906	0.2281	0.070*	
C12	0.43103 (10)	0.19237 (15)	0.11810 (8)	0.0610 (3)	
H12	0.4834	0.1127	0.1160	0.073*	
C13	0.36727 (12)	0.26327 (16)	0.05411 (8)	0.0636 (4)	
H13	0.3767	0.2321	0.0090	0.076*	
C14	0.28910 (11)	0.38117 (15)	0.05734 (7)	0.0578 (3)	
H14	0.2468	0.4311	0.0144	0.069*	
C15	−0.07866 (10)	0.29493 (13)	0.07155 (7)	0.0489 (3)	
C16	−0.10077 (12)	0.17938 (15)	0.01980 (7)	0.0595 (3)	
H16	−0.0431	0.1553	−0.0042	0.071*	
C17	−0.20769 (13)	0.09931 (18)	0.00341 (8)	0.0753 (4)	
H17	−0.2218	0.0210	−0.0313	0.090*	
C18	−0.29321 (13)	0.1351 (2)	0.03829 (11)	0.0859 (5)	
H18	−0.3660	0.0823	0.0269	0.103*	
C19	−0.27106 (13)	0.24912 (18)	0.09009 (11)	0.0851 (5)	
H19	−0.3291	0.2726	0.1140	0.102*	
C20	−0.16407 (11)	0.32979 (16)	0.10748 (9)	0.0663 (4)	
H20	−0.1498	0.4066	0.1430	0.080*	
C21	0.58910 (15)	0.6035 (2)	0.12019 (10)	0.0872 (5)	
H21A	0.6736	0.6041	0.1238	0.131*	
H21B	0.5450	0.5624	0.0733	0.131*	
H21C	0.5752	0.5414	0.1589	0.131*	
N1	0.18861 (7)	0.54311 (10)	0.12879 (5)	0.0475 (2)	
N2	−0.01409 (8)	0.60588 (11)	0.12228 (6)	0.0514 (3)	
N3	0.03525 (9)	0.36584 (12)	0.08787 (6)	0.0559 (3)	
H3A	0.0925 (13)	0.3156 (15)	0.0783 (8)	0.067*	
O1	0.34102 (7)	0.71482 (11)	0.16472 (5)	0.0673 (3)	
O2	0.31895 (9)	0.40317 (12)	0.25304 (5)	0.0712 (3)	
H2A	0.3655 (16)	0.346 (2)	0.2930 (11)	0.107*	
O3	0.54980 (9)	0.75565 (12)	0.12703 (6)	0.0731 (3)	
H3B	0.4742 (18)	0.750 (2)	0.1312 (11)	0.110*	

### Atomic displacement parameters (Å^2^)

**Table:**

	*U*^11^	*U*^22^	*U*^33^	*U*^12^	*U*^13^	*U*^23^
C1	0.0534 (7)	0.0500 (7)	0.0447 (6)	−0.0022 (5)	0.0114 (5)	0.0037 (5)
C2	0.0520 (7)	0.0475 (7)	0.0478 (6)	0.0024 (5)	0.0149 (5)	0.0089 (5)
C3	0.0614 (8)	0.0534 (8)	0.0672 (8)	0.0088 (6)	0.0233 (6)	0.0111 (6)
C4	0.0882 (11)	0.0562 (8)	0.0676 (9)	0.0171 (7)	0.0304 (8)	0.0088 (7)
C5	0.0980 (12)	0.0502 (8)	0.0643 (9)	−0.0023 (7)	0.0246 (8)	−0.0033 (6)
C6	0.0712 (9)	0.0586 (8)	0.0580 (8)	−0.0113 (7)	0.0157 (6)	−0.0035 (6)
C7	0.0442 (6)	0.0549 (7)	0.0482 (7)	−0.0061 (5)	0.0074 (5)	0.0027 (5)
C8	0.0383 (6)	0.0494 (7)	0.0498 (6)	−0.0011 (5)	0.0098 (5)	0.0041 (5)
C9	0.0338 (5)	0.0531 (7)	0.0554 (7)	−0.0019 (5)	0.0121 (5)	0.0008 (5)
C10	0.0382 (6)	0.0602 (7)	0.0555 (7)	−0.0007 (5)	0.0140 (5)	0.0028 (6)
C11	0.0407 (6)	0.0634 (8)	0.0686 (8)	0.0039 (6)	0.0136 (6)	0.0073 (7)
C12	0.0448 (7)	0.0566 (8)	0.0860 (10)	−0.0001 (6)	0.0257 (7)	−0.0024 (7)
C13	0.0612 (8)	0.0689 (9)	0.0664 (9)	−0.0030 (7)	0.0270 (7)	−0.0102 (7)
C14	0.0512 (7)	0.0654 (8)	0.0562 (8)	−0.0004 (6)	0.0140 (6)	0.0022 (6)
C15	0.0389 (6)	0.0494 (7)	0.0553 (7)	−0.0023 (5)	0.0082 (5)	0.0037 (5)
C16	0.0588 (8)	0.0675 (8)	0.0519 (7)	−0.0093 (6)	0.0148 (6)	−0.0031 (6)
C17	0.0729 (10)	0.0792 (10)	0.0674 (9)	−0.0250 (8)	0.0090 (7)	−0.0155 (8)
C18	0.0508 (8)	0.0876 (12)	0.1150 (14)	−0.0237 (8)	0.0160 (9)	−0.0126 (10)
C19	0.0535 (8)	0.0737 (10)	0.1372 (16)	−0.0113 (7)	0.0414 (9)	−0.0175 (10)
C20	0.0490 (7)	0.0605 (8)	0.0927 (11)	−0.0064 (6)	0.0253 (7)	−0.0138 (7)
C21	0.0891 (11)	0.0905 (12)	0.0887 (12)	−0.0085 (9)	0.0360 (9)	−0.0110 (9)
N1	0.0362 (5)	0.0513 (6)	0.0531 (6)	0.0001 (4)	0.0095 (4)	0.0021 (4)
N2	0.0422 (5)	0.0489 (6)	0.0629 (6)	0.0026 (4)	0.0142 (4)	0.0034 (5)
N3	0.0384 (5)	0.0523 (6)	0.0776 (7)	−0.0009 (4)	0.0173 (5)	−0.0092 (5)
O1	0.0433 (5)	0.0739 (6)	0.0811 (7)	−0.0113 (4)	0.0114 (4)	−0.0051 (5)
O2	0.0689 (6)	0.0885 (7)	0.0568 (6)	0.0236 (5)	0.0184 (5)	0.0081 (5)
O3	0.0584 (6)	0.0883 (8)	0.0715 (6)	−0.0126 (5)	0.0163 (5)	−0.0109 (5)

### Geometric parameters (Å, °)

**Table:**

C1—C2	1.3917 (16)	C12—C13	1.3743 (19)
C1—C6	1.4031 (18)	C12—H12	0.9300
C1—C7	1.4502 (17)	C13—C14	1.3822 (18)
C2—N2	1.3879 (15)	C13—H13	0.9300
C2—C3	1.4017 (16)	C14—H14	0.9300
C3—C4	1.3701 (19)	C15—C16	1.3781 (17)
C3—H3	0.9300	C15—C20	1.3796 (17)
C4—C5	1.3840 (19)	C15—N3	1.4086 (14)
C4—H4	0.9300	C16—C17	1.3775 (18)
C5—C6	1.3608 (19)	C16—H16	0.9300
C5—H5	0.9300	C17—C18	1.369 (2)
C6—H6	0.9300	C17—H17	0.9300
C7—O1	1.2244 (13)	C18—C19	1.369 (2)
C7—N1	1.3901 (15)	C18—H18	0.9300
C8—N2	1.2869 (14)	C19—C20	1.3804 (18)
C8—N3	1.3640 (15)	C19—H19	0.9300
C8—N1	1.3969 (13)	C20—H20	0.9300
C9—C14	1.3793 (17)	C21—O3	1.4210 (19)
C9—C10	1.3893 (17)	C21—H21A	0.9600
C9—N1	1.4482 (14)	C21—H21B	0.9600
C10—O2	1.3554 (16)	C21—H21C	0.9600
C10—C11	1.3848 (16)	N3—H3A	0.854 (14)
C11—C12	1.3755 (19)	O2—H2A	0.940 (19)
C11—H11	0.9300	O3—H3B	0.90 (2)
			
C2—C1—C6	120.14 (11)	C14—C13—H13	120.2
C2—C1—C7	119.19 (11)	C9—C14—C13	119.83 (12)
C6—C1—C7	120.67 (11)	C9—C14—H14	120.1
N2—C2—C1	122.47 (11)	C13—C14—H14	120.1
N2—C2—C3	118.96 (11)	C16—C15—C20	119.63 (11)
C1—C2—C3	118.57 (11)	C16—C15—N3	116.96 (11)
C4—C3—C2	120.22 (13)	C20—C15—N3	123.30 (11)
C4—C3—H3	119.9	C17—C16—C15	120.50 (13)
C2—C3—H3	119.9	C17—C16—H16	119.8
C3—C4—C5	120.92 (13)	C15—C16—H16	119.8
C3—C4—H4	119.5	C18—C17—C16	119.95 (14)
C5—C4—H4	119.5	C18—C17—H17	120.0
C6—C5—C4	119.91 (13)	C16—C17—H17	120.0
C6—C5—H5	120.0	C17—C18—C19	119.64 (13)
C4—C5—H5	120.0	C17—C18—H18	120.2
C5—C6—C1	120.22 (12)	C19—C18—H18	120.2
C5—C6—H6	119.9	C18—C19—C20	121.13 (14)
C1—C6—H6	119.9	C18—C19—H19	119.4
O1—C7—N1	120.15 (11)	C20—C19—H19	119.4
O1—C7—C1	124.71 (11)	C15—C20—C19	119.14 (13)
N1—C7—C1	115.14 (10)	C15—C20—H20	120.4
N2—C8—N3	121.42 (10)	C19—C20—H20	120.4
N2—C8—N1	124.48 (11)	O3—C21—H21A	109.5
N3—C8—N1	114.10 (10)	O3—C21—H21B	109.5
C14—C9—C10	120.95 (11)	H21A—C21—H21B	109.5
C14—C9—N1	120.83 (10)	O3—C21—H21C	109.5
C10—C9—N1	118.20 (10)	H21A—C21—H21C	109.5
O2—C10—C11	124.12 (11)	H21B—C21—H21C	109.5
O2—C10—C9	117.49 (11)	C7—N1—C8	121.07 (10)
C11—C10—C9	118.39 (12)	C7—N1—C9	117.90 (9)
C12—C11—C10	120.59 (12)	C8—N1—C9	120.82 (9)
C12—C11—H11	119.7	C8—N2—C2	117.48 (10)
C10—C11—H11	119.7	C8—N3—C15	128.13 (10)
C13—C12—C11	120.68 (12)	C8—N3—H3A	114.4 (9)
C13—C12—H12	119.7	C15—N3—H3A	117.4 (9)
C11—C12—H12	119.7	C10—O2—H2A	110.0 (11)
C12—C13—C14	119.52 (13)	C21—O3—H3B	107.3 (12)
C12—C13—H13	120.2		
			
C6—C1—C2—N2	177.94 (11)	N3—C15—C16—C17	176.81 (12)
C7—C1—C2—N2	−1.67 (17)	C15—C16—C17—C18	0.5 (2)
C6—C1—C2—C3	−1.20 (17)	C16—C17—C18—C19	−0.9 (3)
C7—C1—C2—C3	179.18 (11)	C17—C18—C19—C20	0.5 (3)
N2—C2—C3—C4	−179.27 (11)	C16—C15—C20—C19	−0.8 (2)
C1—C2—C3—C4	−0.10 (18)	N3—C15—C20—C19	−176.97 (13)
C2—C3—C4—C5	1.5 (2)	C18—C19—C20—C15	0.4 (3)
C3—C4—C5—C6	−1.6 (2)	O1—C7—N1—C8	−177.48 (10)
C4—C5—C6—C1	0.2 (2)	C1—C7—N1—C8	3.11 (16)
C2—C1—C6—C5	1.14 (18)	O1—C7—N1—C9	7.77 (16)
C7—C1—C6—C5	−179.25 (12)	C1—C7—N1—C9	−171.63 (9)
C2—C1—C7—O1	178.64 (11)	N2—C8—N1—C7	−0.53 (17)
C6—C1—C7—O1	−0.98 (18)	N3—C8—N1—C7	178.81 (10)
C2—C1—C7—N1	−1.99 (16)	N2—C8—N1—C9	174.06 (11)
C6—C1—C7—N1	178.40 (10)	N3—C8—N1—C9	−6.60 (15)
C14—C9—C10—O2	179.84 (11)	C14—C9—N1—C7	−104.81 (13)
N1—C9—C10—O2	−1.35 (16)	C10—C9—N1—C7	76.38 (13)
C14—C9—C10—C11	−0.32 (17)	C14—C9—N1—C8	80.43 (13)
N1—C9—C10—C11	178.49 (10)	C10—C9—N1—C8	−98.38 (13)
O2—C10—C11—C12	178.59 (12)	N3—C8—N2—C2	177.48 (11)
C9—C10—C11—C12	−1.24 (17)	N1—C8—N2—C2	−3.23 (17)
C10—C11—C12—C13	1.56 (19)	C1—C2—N2—C8	4.31 (16)
C11—C12—C13—C14	−0.29 (19)	C3—C2—N2—C8	−176.55 (10)
C10—C9—C14—C13	1.57 (18)	N2—C8—N3—C15	−4.5 (2)
N1—C9—C14—C13	−177.21 (11)	N1—C8—N3—C15	176.17 (11)
C12—C13—C14—C9	−1.26 (19)	C16—C15—N3—C8	153.97 (13)
C20—C15—C16—C17	0.4 (2)	C20—C15—N3—C8	−29.8 (2)

### Hydrogen-bond geometry (Å, °)

**Table:**

*D*—H···*A*	*D*—H	H···*A*	*D*···*A*	*D*—H···*A*
O2—H2A···O3^i^	0.940 (19)	1.74 (2)	2.6775 (14)	173.8 (17)
C11—H11···O1^i^	0.93	2.59	3.3781 (17)	143.
O3—H3B···O1	0.90 (2)	1.85 (2)	2.7237 (14)	164.1 (18)

Symmetry codes: (i) −*x*+1, *y*−1/2, −*z*+1/2.
